# The D-Dimer to Troponin Ratio Is a Novel Marker for the Differential Diagnosis of Thoracic Acute Aortic Syndrome from Non-ST Elevation Myocardial Infarction

**DOI:** 10.3390/jcm12093054

**Published:** 2023-04-22

**Authors:** Minsik Lee, Yong Won Kim, Dayeon Lee, Tae-Youn Kim, Sanghun Lee, Jun Seok Seo, Jeong Hun Lee

**Affiliations:** 1 Department of Emergency Medicine, Dongguk University Ilsan Hospital, Dongguk University College of Medicine, Goyang 10326, Republic of Korea; 2 Department of Emergency Medicine, Kangwon National University College of Medicine, Chuncheon 24341, Republic of Korea

**Keywords:** acute aortic syndrome, non-ST elevation myocardial infraction, differential diagnosis, D-dimer, troponin T

## Abstract

Background: Thoracic acute aortic syndrome (AAS) and non-ST elevation myocardial infarction (NSTEMI) have similar clinical presentations, making them difficult to differentiate. This study aimed to identify useful biomarkers for the differential diagnosis of thoracic AAS and NSTEMI. Methods: This was a retrospective observational study. Participants: consecutive adult patients who visited the emergency department for acute chest pain between January 2015 and December 2021 diagnosed with thoracic AAS or NSTEMI. Clinical variables, including D-dimer (μg/mL) and high-sensitivity troponin T (ng/mL, hs-TnT) levels, were compared between the groups. Results: A total of 52 (30.1%) and 121 (69.9%) patients were enrolled in the thoracic AAS and NSTEMI groups, respectively. Logistic regression analysis revealed that the D-dimer to hs-TnT (D/T) ratio (odds ratio (OR), 1.038; 95% confidence interval (CI), 1.020–1.056; *p* < 0.001) and the thrombolysis in myocardial infarction (TIMI) score (OR, 0.184; 95% CI, 0.054–0.621; *p* = 0.006) were associated with thoracic AAS. The D/T ratio had an area under the receiver operating characteristic curve (AUC) of 0.973 (95% CI, 0.930–0.998), and the optimal cutoff value was 81.3 with 91.4% sensitivity and 96.2% specificity. The TIMI score had an AUC of 0.769 (95% CI, 0.644–0.812), and the optimal cutoff value was 1.5 with 96.7% sensitivity and 38.5% specificity. Conclusion: the D/T ratio may be a simple and useful parameter for differentiating thoracic AAS from NSTEMI.

## 1. Introduction

Thoracic acute aortic syndrome (AAS) and non-ST elevation myocardial infarction (NSTEMI) commonly present with acute chest pain as the chief symptom. Both are fatal acute diseases that require early diagnosis and treatment. However, diagnosis of these diseases by primary evaluation alone is not easy and often requires advanced imaging modalities or invasive interventions such as computed tomography angiography (CTA) or percutaneous angiography [1,2]. These additional tests are expensive, time-consuming, and carry risks (such as radiation exposure and contrast-induced nephropathy); therefore, researchers have developed and recommend the use of risk-scoring tools (such as the acute aortic dissection risk score (ADD-RS) and the thrombolysis in myocardial infarction (TIMI) score) for each disease to minimize unnecessary testing and risk stratification [3,4]. However, these risk scoring tools may not facilitate differentiating between the diseases because they are clinically used as screening tools with high sensitivity and low specificity owing to the lethal characteristics of both diseases.

As a biomarker, troponin is associated with acute myocardial infarction (AMI), whereas D-dimer is associated with AAS. Therefore, these biomarkers can be used to diagnose or exclude each disease [2,5]. However, troponin, a cardiac-specific biomarker, is elevated not only in AMI, but also in other diseases, including AAS [6,7]. Similarly, D-dimer is elevated in various conditions (such as pulmonary embolism, AAS, and AMI) according to secondary fibrinolysis and intravascular activation of the coagulation system [8,9]. Therefore, differentiating between AAS and NSTEMI based on positive troponin or D-dimer levels may not be useful. Increases in the troponin and D-dimer levels may depend on the disease. Moreover, pulmonary embolism (PE) and NSTEMI can be differentiated using the D-dimer to troponin (D/T) ratio rather than using the absolute value of each biomarker [10].

We aimed to verify the usefulness of the ADD-RS, TIMI score, troponin level, D-dimer level, and D/T ratio in differentiating between AAS and NSTEMI.

## 2. Method

### 2.1. Study Design and Participants

This single-center retrospective study was conducted in adults aged ≥ 18 years who presented with acute chest pain to the emergency room (ER) and were diagnosed with thoracic AAS or NSTEMI between January 2015 and December 2021. The following patients were excluded: those with trauma-related conditions, those transferred to another facility, those who did not visit the ER within 24 h of symptom onset, and those whose initial blood tests did not include D-dimer or high-sensitivity troponin T (hs-TnT) levels.

During the study, cardiologists and thoracic surgeons performed percutaneous coronary intervention and aortic surgery for 24 h at our institution. In patients with acute chest pain in the emergency department, the plasma hs-TnT and D-dimer levels were routinely measured during initial blood sampling. Aortic CTA was selectively performed in patients with an ADD-RS ≥ 1 under the direction of the attending emergency physicians for differential diagnosis. In patients with a TIMI score ≥ 2, the attending cardiologist selectively performed emergency or elective percutaneous coronary angiography after comprehensively assessing the condition.

### 2.2. D-Dimer and Troponin T Measurement

The D-dimer levels were measured by means of a turbidimetric immunoassay using Sysmex CS-5100 (Sysmex, Kobe, Japan), and the reference value was <0.55 μg/mL. The hs-TnT levels were measured by means of the sandwich principle and an electrochemiluminescence immunoassay using a Cobas C8000 modular analyzer (Roche, Mannheim, Germany), and the reference value was <0.014 ng/mL. If the D-dimer value was <0.24 μg/mL or >33.60 μg/mL according to the laboratory equipment, we entered 0.24 μg/mL or 33.60 μg/mL, respectively, during data collection. If the hs-TnT value was <0.003 ng/mL or >10.000 ng/mL according to the laboratory equipment, we entered 0.003 ng/mL or 10 ng/mL, respectively, during data collection. For each biomarker, values higher than the reference value were considered positive. The D/T ratios were calculated retrospectively.

### 2.3. Diagnosis of AAS and NSTEMI

Thoracic AAS was confirmed by CTA. CTA images were obtained using a SOMATOM Definition AS CT scanner (Siemens, Erlangen, Germany) and read by a radiologist. The aortic dissection (AD) protocol requires the injection of a contrast material at a rate of 4 mL/s, 100 mL of a contrast agent, a section thickness of 2 mm, and bolus tracking at the descending thoracic aorta at the level of the carina from 1 cm superior to the lung apices to the aortic bifurcation (level of S1). One of the following CTA-based diagnoses was thoracic AAS: AD, intramural hematoma, penetrating aortic ulcer, or aortic aneurysm of the thoracic aorta. AMI without ST segment elevation on electrocardiography (ECG) was diagnosed as NSTEMI. AMI was diagnosed based on the fourth universal definition of myocardial infarction [11].

### 2.4. Data Collection

Clinical data, including age, sex, body mass index (BMI), medical history (hypertension, diabetes, and smoking), initial clinical presentation (primary complaint, symptom onset to the visit interval, initial vital signs, ADD-RS, TIMI score, initial (sampled within at least 1 h following the emergency department visit) plasma D-dimer level (μg/mL), initial plasma hs-TnT level (ng/mL), ECG findings, aortic CTA findings, coronary angiography findings, final diagnosis, date of admission, and mortality, were derived from electronic medical records. BMI was calculated by dividing weight (in kilograms) by the square of height (in meters). Smokers were defined as current smokers or ex-smokers. ST changes were defined as ST segment elevation (≥0.1 mV at the J-point in two contiguous leads in all the leads other than leads V_2_–V_3_ where flowing cut points apply: ≥0.2 mV in men ≥ 40 years; ≥0.25 mV in men < 40 years; ≥0.15 mV in women) or ST segment depression (≥0.05 mV at the J-point in two contiguous leads). T changes were defined as inverted T wave (≥0.1 mV in two contiguous leads with the R/S ratio > 1), biphasic T wave (rise and then fall pattern), or hyperacute T wave (wide base, round peak, and large relative to the preceding QRS complex). Mortality was defined as death during hospitalization or discharge from the hospital. We calculated the ADD-RS and the TIMI score as shown in Table 1 [12,13].

### 2.5. Statistical Analysis

The study variables were compared between the thoracic AAS and NSTEMI groups. Nominal data were calculated as percentages based on the frequency of occurrence and compared using the chi-squared test or Fisher’s exact test as appropriate. The continuous variables were presented as the median values (interquartile range (IQR)) and compared using the Mann–Whitney test. We performed multivariate logistic regression analysis for the clinical variables associated with AAS. Moreover, we conducted an area under the receiver operating characteristic (ROC) curve analysis to identify the optimal cutoff value, sensitivity, and specificity of the biomarkers and the clinical score for differentiating the diseases. The resulting odds ratios (ORs) are presented with 95% confidence intervals (95% CIs). Statistical significance was defined as a two-sided *p*-value of < 0.05. All statistical analyses were performed using the IBM Statistical Package for the Social Sciences (SPSS) software (version 24.0; SPSS Inc., Chicago, IL, USA).

## 3. Results

Among the patients presenting to the ER with chest pain, 317 were diagnosed with thoracic AAS (n = 116) or NSTEMI (n = 201). Following the exclusion of those with trauma (n = 5), those transferred from the ED (n = 21), those who did not undergo laboratory tests for D-dimer or hs-TnT (n = 80), and those who had the symptom onset time of > 24 h prior (n = 38), 173 patients (thoracic AAS, n = 52; NSTEMI, n = 121) were enrolled in this analysis.

Table 2 summarizes the patients’ general characteristics. The thoracic AAS group had more men (84.6% vs. 69.4%, *p* = 0.039), higher D-dimer levels (10.71 (3.61–33.60) vs. 0.36 (0.24–0.89) μg/mL, *p* < 0.001), more positive D-dimer levels (96.2% vs. 39.7%, *p* < 0.001), a higher D/T ratio (826.2 (246.3–2094.6) vs. 9.4 (2.0–25.5), *p* < 0.001), longer hospitalization (16 (10–21) vs. 5 (4–7) days, *p* < 0.001), and higher mortality (19.2% vs. 2%, *p* < 0.001) than the NSTEMI group. The NSTEMI group had a history of diabetes (5.8% vs. 29.8%, *p* = 0.001), a higher number of smokers (42.3% vs. 59.5%, *p* = 0.046), higher TIMI scores (2 (1–3) vs. 3 (2–4), *p* < 0.001), higher hs-TnT levels (0.012 (0.007–0.023) vs. 0.066 (0.020–0.300) ng/mL, *p* < 0.001), and more positive hs-TnT levels (83.5% vs. 38.5%, *p* < 0.001) than the thoracic AAS group. There were no significant differences in the remaining clinical variables between the groups.

In the thoracic AAS group (n = 52), 29 (55.8%) patients had lesions in the ascending aorta. The most common type of AAS was AD (n = 45), followed by intramural hematoma (n = 26), aortic aneurysm (n = 7), and penetrating aortic ulcers (n = 5). Of the 121 patients with NSTEMI (n = 121), 111 (91.7%) underwent percutaneous coronary angiography, and coronary thrombotic lesions were confirmed in 100 patients (82.6%). Table 3 summarizes the cases (n = 2) with false-negative results in the D-dimer approach for ruling out thoracic AAS and cases (n = 2) with ST elevation in thoracic AAS.

Table 4 presents univariate and multivariate regression analyses of the factors associated with AAS. The multivariate analysis revealed that the TIMI score (OR, 0.184; 95% CI, 0.054–0.621, *p* = 0.006) and the D/T ratio (OR, 1.038; 95% CI, 1.020–1.056, *p* < 0.001) were associated with thoracic AAS.

**Figure 1:**
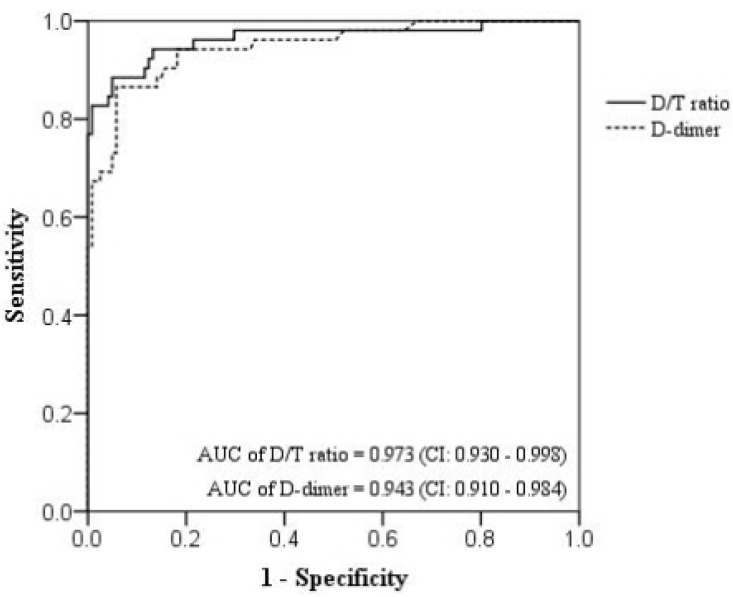
Receiver operating characteristic curve of the D/T ratio and D-dimer for the detection of thoracic AAS. D/T ratio, D-dimer/high-sensitivity troponin T ratio; NSTEMI, non-ST elevation myocardial infarction; AUC, area under the receiver operating characteristic curve.

In differentiating thoracic AAS from NSTEMI, the D/T ratio had an area under the receiver operating characteristic curve (AUC) of 0.973 (95% CI, 0.930–0.998), with the optimal cutoff value, sensitivity, and specificity of 81.3, 91.4%, and 96.2%, respectively (Figure 1). The D-dimer value had an AUC of 0.943 (95% CI, 0.910–0.984), with the optimal cutoff value, sensitivity, and specificity of 1.185 μg/mL, 94.2%, and 91.8%, respectively (Figure 1).

**Figure 2:**
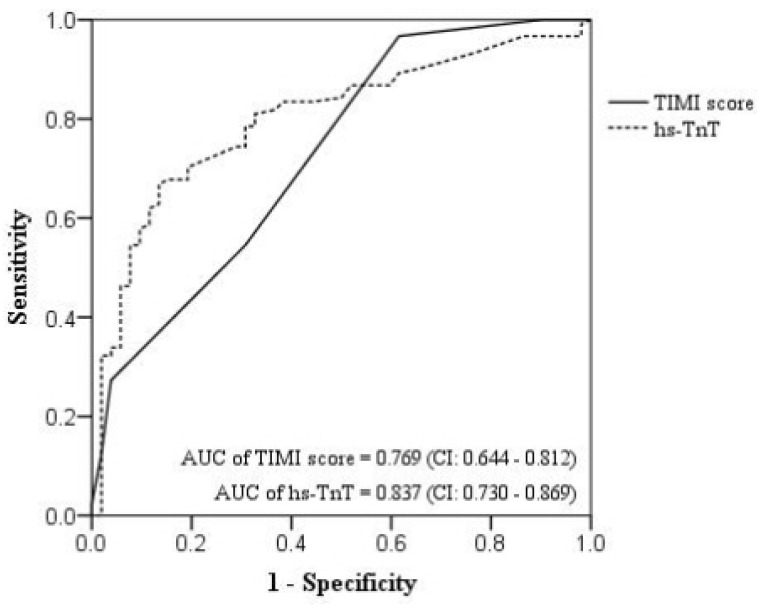
A receiver operating characteristic curve of the TIMI score and hs-TnT for the detection of NSTEMI. TIMI, thrombolysis in myocardial infarction; hs-TnT, high-sensitivity troponin T; NSTEMI, non-ST elevation myocardial infarction; AUC, area under the receiver operating characteristic curve.

In differentiating NSTEMI from thoracic AAS, the TIMI score had an AUC of 0.769 (95% CI, 0.644–0.812), with the optimal cutoff value, sensitivity, and specificity of 1.5, 96.7%, and 38.5%, respectively (Figure 2). The hs-TnT level had an AUC of 0.837 (95% CI, 0.730–0.869), with the optimal cutoff value, sensitivity, and specificity of 0.025 ng/mL, 71.1%, and 78.8%, respectively (Figure 2).

## 4. Discussion

Currently, the symptoms, signs, ECG, chest radiography, and cardiac biomarkers are the main criteria for the initial evaluation of chest pain [1,2,14]. The most common symptoms of thoracic AAS and NSTEMI comprise acute chest pain, whereas other nonspecific symptoms (e.g., diaphoresis, syncope, dyspnea, and abdominal pain) could present similarly. Despite marginal differences in the details of chest pain in both diseases (AAS usually presents with tearing pain and/or pain radiating to the neck or the left shoulder while NSTEMI usually presents with pressure pain and/or pain radiating to the back or the extremities), the symptoms may be insufficient for an alternative diagnosis because they are often subjective and ambiguous. Signs of thoracic AAS and NSTEMI are often nonspecific. Certain specific findings that likely represent the site of involvement in AAS (e.g., early diastolic murmur with aortic regurgitation, stroke-like signs, and bilateral blood pressure differences) can provide clues leading to the suspicion of AAS. However, the absence of these findings does not indicate that NSTEMI is the most likely diagnosis. ECG findings of thoracic AAS and NSTEMI can be relatively normal or initially nondiagnostic [15,16]. Nonspecific findings suggestive of NSTEMI include transient ST elevation, ST depression, or recent T wave inversions [17]. Occasionally, ST segment elevation may occur when AAS involves a coronary artery lesion, which can confound the diagnosis [18]. Chest X-ray can detect abnormalities in the aortic contour or size of the thoracic AAS [19]. However, simple chest radiography has a low sensitivity of approximately 64% and a specificity of approximately 86% for aortic disease, making it difficult to use as a confirmative diagnostic tool. Therefore, it is difficult to distinguish between the two groups based on primary assessments commonly performed in the ER.

Among these biomarkers, troponin and D-dimer are commonly used to screen patients with chest pain in the ER because they can be obtained quickly. Although both tests are often used clinically as positive tests with cutoff values greater than the 99th percentile, these biomarkers can be positive for both AAS and AMI [6,7,8,9]. Similarly, in our study, false-positive hs-TnT findings occurred in 39% of the AAS cases, and false-positive D-dimer findings occurred in 40% of the NSTEMI cases. Therefore, it is difficult to use these biomarkers to differentiate between AAS and NSTEMI in a dichotomous manner (positive vs. negative) as suggested by the guidelines for each disease. Nevertheless, troponin levels were higher in AMI than in other diseases, whereas D-dimer levels were higher in AAS than in AMI [20,21]. In our study, D-dimer (AUC of 0.943) displayed an excellent diagnostic accuracy for thoracic AAS and hs-TnT (AUC of 0.837) displayed a good diagnostic accuracy for NSTEMI. Therefore, these biomarkers can be used to quantitatively differentiate thoracic AAS from NSTEMI when both the troponin and D-dimer levels are positive. In addition, as shown in our study, the use of the D/T ratio (AUC = 0.973) improved the diagnostic accuracy.

For risk stratification, ADD-RS and TIMI are commonly used for AAS and NSTEMI, respectively [3,4]. Chest pain, hypotension, ECG changes, and positive troponin levels can be present in both AAS and NSTEMI and are commonly included in both risk score factors. Therefore, both the ADD-RS and the TIMI score could be elevated in both diseases. In our study, the interquartile range of each risk score for both diseases ranged from low to intermediate. In the multivariate analysis, TIMI was related to NSTEMI, whereas ADD-RS was not statistically related to NSTEMI or AAS. This result may be attributed to the total score range of TIMI (0–7) being broader than that of ADD-RS (0–3), with more factors contributing to the score included in TIMI being specific to coronary artery disease.

Physicians can consider a noninvasive bedside examination, namely transthoracic echocardiography (TTE), for the differential diagnosis of acute chest pain [22]. Specific TTE findings in AAS include visualization of the proximal dissection of the aorta. TTE for AAS displays a high specificity but a relatively low sensitivity because it cannot scan the entire aorta, but only a segment of the proximal aorta. The specific TTE finding in AMI is visualization of regional wall motion abnormalities in the left ventricular wall. TTE for AMI has a high sensitivity but a relatively low specificity. This is because regional wall motion abnormalities can be observed in other conditions such as prior MI or conduction abnormalities. Nonspecific findings, such as pericardial effusion and/or recent-onset valve insufficiency, can be observed in both AAS and AMI through TTE. TTE may help reduce the clinical suspicion in patients with chest pain; however, it is not essential for the diagnosis of AAS or AMI. In addition, the effectiveness of ultrasound depends on the skill of the operator, which restricts its universal use in various medical environments owing to the need for equipment and manpower.

Few studies have been conducted on the D/T ratio; however, one study reported that the D/T ratio is useful for differentiating between acute PE and NSTEMI [10]. The mentioned study measured troponin I (TnI), which is different from our study. Similarly to the results of our study, the diagnostic accuracy of the D/T ratio (AUC, 0.951; sensitivity, 93.3%; specificity, 86.6%) was better than that of D-dimer (AUC, 0.860; sensitivity, 81.1%; specificity, 70.2%) or TnI (AUC, 0.875; sensitivity, 80.6%; specificity, 78.9%). However, unlike thoracic AAS or NSTEMI in which chest pain is the main symptom, the main symptom of PE is dyspnea. Previous studies have shown that the misdiagnosis rate of aortic dissection is as high as 39%, with the most common incorrect initial diagnosis being acute coronary syndrome. Delays in accurate diagnosis and inappropriate treatment with anticoagulants can lead to excessive bleeding and prolonged hospitalization [23]. Therefore, most clinicians should be more concerned about differentiating between thoracic AAS and NSTEMI than between acute PE and NSTEMI. Nonetheless, in clinical scenarios where the D/T ratio is high, optional aortic CTA as confirmatory imaging can be used to diagnose both PE and AAS.

Unlike troponin, which gradually normalizes after the acute phase of MI, the D-dimer levels may remain elevated in chronic aortic diseases because of the coagulation response to an ongoing thrombotic process [24]. Therefore, the use of the D/T ratio may be limited if a chronic aortic disease exists prior to NSTEMI. Nevertheless, in a clinical setting, if a patient with a chronic aortic disease presents with acute chest pain, clinicians prioritize approaches to differentiate AAS regardless of the D/T ratio, even if the final diagnosis is NSTEMI. Additionally, if the chronic aortic disease is unknown and is diagnosed incidentally through a test performed because of a high D/T ratio, this should be an important consideration when selecting treatment options. AD of the proximal aorta is often accompanied by myocardial ischemia owing to coronary malperfusion, which can affect the D/T ratio [25]. Previous studies have reported signs of AMI in 5% of patients with AD and in 9.3% of patients with Stanford type A AD [26,27]. However, AD has relatively high D-dimer levels in AAS, so we believe that a high D/T ratio is likely to be maintained [5]. In addition, coronary malperfusion due to IMH may occur in rare cases [28]. Future studies on the diagnostic accuracy of the D/T ratio in these cases are warranted.

## 5. Limitations

This study has some limitations. First, this was a retrospective study, and the onset time differed between the groups. However, the median value of the symptom-based onset time was approximately 3.2 h and 3.0 h for AAS and NSTEMI, respectively, without a significant difference. Even if a prospective study was conducted, it would be difficult to accurately match the onset times of these two acute diseases. Second, the study was conducted at a single center and requires re-evaluation in a different medical environment. Troponin is a time-dependent marker that does not increase in the early phase, but increases gradually. In contrast, the D-dimer levels increase in the early phase. Therefore, hospital accessibility may have generated different results. This warrants further studies to measure serial D-dimer and troponin levels to determine gradual changes in the D/T ratio. Third, the degree of increase in troponin levels may depend on the troponin type. Contemporary cardiac troponin levels may not increase within the first 2–4 h of symptom onset; however, the high-sensitivity troponin assays used in our study had the advantage of increasing earlier. Further studies are required to determine the D/T ratio according to the troponin type. Fourth, we did not perform a subgroup analysis according to disease severity or uniformity of the AAS. The type of AAS or size of the myocardial infarction may affect the increase in the D-dimer and troponin levels. However, there were insufficient cases for performing these analyses. Moreover, not all patients had an infarct lesion confirmed by coronary angiography.

## 6. Conclusions

The D-dimer level, the D/T ratio, and the TIMI score are useful clinical factors for the differential diagnosis between thoracic AAS and NSTEMI. A high D/T ratio is a novel marker with the highest diagnostic accuracy in discriminating thoracic AAS from NSTEMI. The D/T ratio can be obtained easily and quickly; therefore, its use with a comprehensive diagnostic approach for acute chest pain will reduce diagnostic delays or misdiagnosis and can be used as evidence to select an appropriate confirmation test. Further prospective studies are required to validate this D/T ratio.

## Figures and Tables

**Table 1:** ADD-RS and TIMI score calculation.

Criteria		Points
ADD-RS		
Any high-risk condition	Marfan syndrome	1
	Family history of aortic disease	
	Known aortic valve disease	
	Previous aortic manipulation	
	Known thoracic aortic aneurysm	
Any high-risk pain feature	Abrupt onset of pain	1
	Severe pain intensity	
	Ripping or tearing pain	
Any high-risk exam feature	Pulse deficit or SBP differential	1
	Focal neurologic deficit	
	New aortic insufficiency murmur (or sonographic)	
	Hypotension/shock state	
Total score		0 to 3
TIMI score		
Age, ≥65 years		1
≥3 CAD risk factors		1
Known CAD (stenosis ≥ 50%)		1
Use of aspirin in the last seven days		1
Severe anginal symptoms (e.g., two episodes in 24 h)		1
ECG ST change ≥ 0.5 mm		1
Positive hs-TnT		1
Total score		0 to 7

ADD-RS, aortic dissection detection risk score; SBP, systolic blood pressure; TIMI, thrombolysis in myocardial infarction; CAD, coronary artery disease; ECG, electrocardiography; hs-TnT, high-sensitivity troponin T.

**Table 2:** Comparison of the general characteristics between the thoracic AAS and NSTEMI groups.

	Total (n = 173)	Thoracic AAS (n = 52)	NSTEMI (n = 121)	*p*
Age (years)	65 (55–80) *	66 (54–76) *	64 (55–82) *	0.445
Males, n (%)	128 (74.0)	44 (84.6)	84 (69.4)	0.039
Body mass index	24.0 (22.0–27.0) *	25.0 (23.0–28.0) *	24.0 (22.0–26.0) *	0.064
Medical history				
Diabetes, n (%)	39 (22.5)	3 (5.8)	36 (29.8)	0.001
Hypertension, n (%)	104 (60.1)	33 (63.5)	71 (58.7)	0.614
Smokers, n (%)	94 (54.3)	22 (42.3)	72 (59.5)	0.046
Pack-years among the smokers	30 (15–40) *	30 (19–35) *	30 (10–40) *	0.537
Interval between the symptom onset and the visit (h)	3.0 (1.0–7.0) *	3.2 (2.0–5.6) *	3.0 (1.0–9.0) *	0.753
Initial vital signs				
SBP (mm Hg)	138 (114–161) *	138 (116–158) *	137 (113–164) *	0.631
DBP (mm Hg)	78 (67–94) *	73 (63–91) *	81 (69–95) *	0.066
HR (rate/min)	76 (65–89) *	74 (62–86) *	77 (66–90) *	0.224
RR (rate/min)	20 (18–20) *	20 (18–20) *	20 (19–20) *	0.353
BT (°C)	36.4 (36.0–36.7) *	36.4 (36.0–36.7) *	36.4 (36.0–36.7) *	0.750
SpO_2_ (%)	99.0 (98.0–100.0) *	99.0 (98.0–99.0) *	99.0 (98.0–100.0) *	0.173
EKG finding				0.233
No ST or T change, n (%)	97 (56.1)	35 (67.3)	62 (51.2)	
ST change, n (%)	20 (11.6)	3 (5.8)	17 (14.0)	
T change, n (%)	37 (21.4)	9 (17.3)	28 (23.1)	
ST and T change, n (%)	19 (11.0)	5 (9.6)	14 (11.6)	
ADD-RS	1 (1–1) *	1 (1–1) *	1 (1–1) *	0.001
TIMI score	2 (2–3) *	2 (1–3) *	3 (2–4) *	<0.001
D-dimer (μg/mL)	0.74 (0.26–3.76) *	10.71 (3.61–33.60) *	0.36 (0.24–0.89) *	<0.001
Positive D-dimer, n (%)	98 (56.6)	50 (96.2)	48 (39.7)	<0.001
hs-TnT (ng/mL)	0.034 (0.011–0.184) *	0.012 (0.007–0.023) *	0.066 (0.020–0.300) *	<0.001
Positive hs-TnT, n (%)	121 (69.9)	20 (38.5)	101 (83.5)	<0.001
D/T ratio	18.9 (4.0–146.5) *	826.2 (246.3–2094.6) *	9.4 (2.0–25.5) *	<0.001
HD	6 (4–14) *	16 (10–21) *	5 (4–7) *	<0.001
Mortality, n (%)	12 (6.9)	10 (19.2)	2 (1.7)	<0.001

* Median (interquartile range); AAS, acute aortic syndrome; NSTEMI, non-ST elevation myocardial infarction; SBP, systolic blood pressure; DBP, diastolic blood pressure; HR, heart rate; RR, respiratory rate; BT, body temperature; SpO_2_, peripheral oxygen saturation; ADD-RS, aortic dissection detection risk score; TIMI, thrombolysis in myocardial infarction; hs-TnT, high-sensitivity troponin T; D/T ratio, D-dimer to hs-TnT ratio; HD, hospital day; ICU, intensive care unit.

**Table 3:** Clinical presentation of STEMI mimics and false-negative D-dimer cases in the AAS group.

Case		Clinical Description	Symptom Onset	ECG	TIMI Score	ADD-RS	D-Dimer (μg/mL)	hs-TnT (ng/mL)	D/T Ratio	Type of AAS
STEMI mimics	1	72/F, pressure chest pain, history of HTN, dyslipidemia, low BP (SBP 91/DBP 49) at ER visit	2 h ago	Inferior lead ST elevation	3	1	33.60	0.008	4200.0	AD in the ascending thoracic aorta, IMH in the entire thoracic aorta
	2	62/M, ripping chest pain, history of HTN	4 h ago	Inferolateral lead ST elevation	2	1	3.13	0.016	195.0	AD from the sinus of valsalva to the ascending aorta
False negative D-dimer	1	56/M, sqeezing chest pain, history of HTN, DM, smoking, high BP (SBP 171/DBP 113) at ER visit	0.5 h ago	No ST or T change	2	1	0.34	0.003	113.3	IMH in the descending thoracic aorta
	2	57/M, ripping chest pain, history of HTN, high BP (SBP 158/DBP 93) at ER visit	5 h ago	No ST or T change	3	1	0.26	0.006	43.3	IMH in the entire thoracic aorta, PAU in the aortic arch

STEMI, ST-elevation myocardial infarction; AAS, acute aortic syndrome; ECG, electrocardiogram; TIMI, thrombolysis in myocardial infarction; ADD-RS, aortic dissection detection risk score; hs-TnT, high-sensitivity troponin T; D/T ratio, D-dimer to hs-TnT ratio; HTN, hypertension; BP, blood pressure; ER, emergency room; AD, aortic dissection; DM, diabetes mellitus; IMH, intramural aortic hematoma; PAU, penetrating aortic ulcer.

**Table 4:** Univariate and multivariate logistic regression of the clinical factors associated with acute aortic syndrome.

Clinical Factors	Univariate			Multivariate		
	ODDS Ratio	95% CI	*p*	Odds Ratio	95% CI	*p*
Male sex	2.423	1.039–5.650	0.041	0.097	0.006–1.546	0.099
Past history of diabetes	0.145	0.042–0.494	0.002	0.118	0.002–6.668	0.299
Smoker	0.499	0.258–0.965	0.039	0.437	0.061–3.149	0.411
ADD-RS	4.794	1.761–13.047	0.002	0.667	0.055–8.104	0.751
TIMI score	0.387	0.260–0.577	<0.001	0.184	0.054–0.621	0.006
D-dimer (μg/mL)	2.196	1.639–2.941	<0.001			
Positive D-dimer, n (%)	38.021	8.834–163.638	<0.001			
hs-TnT (ng/mL)	0.450	0.149–1.362	0.158			
Positive hs-TnT, n (%)	0.124	0.059–0.258	<0.001			
D/T ratio	1.024	1.013–1.034	<0.001	1.038	1.020–1.056	<0.001

CI, confidence interval; BP, blood pressure; ER, emergency room; ADD-RS, aortic dissection detection risk score; TIMI, thrombolysis in myocardial infarction; D/T ratio, D-dimer to high-sensitivity troponin T ratio. Multivariate logistic regression analysis included parameters showing significant differences in the univariate analysis (*p* < 0.05), except for D-dimer, positive D-dimer, and positive hs-TnT, which directly affected the D/T ratio.

## Data Availability

The data used to support the findings of this study are available from the corresponding author upon request.
